# Application of fractional exhaled nitric oxide and nasal nitric oxide in the evaluation of asthma control

**DOI:** 10.3389/fped.2025.1567548

**Published:** 2025-07-07

**Authors:** Yucong Ma, Wenying Lin, Haoqi Zheng, Yang Wang, Jingjing Cui, Li Liu

**Affiliations:** ^1^ Department of Pediatric Respiratory, Children’s Medical Center, The First Hospital of Jilin University, Changchun, China; ^2^ Department of Pediatrics, Yanbian University Hospital, Yanji, Jilin, China; ^3^ Department of Rheumatology and Clinical Immunology, Tianjin Children's Hospital, Tianjin, China

**Keywords:** fractional exhaled nitric oxide (FENO), fractional nasally exhaled nitric oxide (FnNO), asthma control test (ACT), asthma, pulmonary function

## Abstract

**Background:**

Asthma is the most common chronic respiratory disease in childhood, and effective control of airway inflammation is crucial in its management. Fractional exhaled nitric oxide (FeNO) and fractional nasally exhaled nitric oxide (FnNO) are non-invasive biomarkers that reflect airway inflammation. This study aimed to evaluate the role of FeNO and FnNO in assessing asthma control status and to explore their correlation with pulmonary function parameters in pediatric patients.

**Methods:**

This was a retrospective observational study. A total of 88 children with asthma were classified into three groups based on Childhood Asthma Control Test (C-ACT) scores: the control group, the partial control group, and the poor control group. FeNO, FnNO, and pulmonary function tests were measured and compared across the three groups. The correlation between FeNO/FnNO levels and pulmonary function indices was also analyzed.

**Results:**

The levels of FeNO and FnNO were significantly higher in the poor control group compared with those in the partial control and the control groups (*p* < 0.05). As asthma control improved, forced vital capacity (FVC) showed a statistically significant increase. The forced expiratory volume in one second (FEV₁), maximal expiratory flow at 50% of FVC (MEF_50%_), and mean mid-expiratory flow (MMEF) values in the poor control group were significantly lower than those in the other two groups, and PEF was significantly reduced compared with that in the control group. However, no significant correlations were found between FeNO or FnNO levels and any of the pulmonary function parameters.

**Conclusion:**

Although FeNO and FnNO levels differed significantly among asthma control groups, no significant correlation was observed between these biomarkers and pulmonary function parameters. These findings suggest that FeNO and FnNO should be used together to assess asthma control status, but they may not directly reflect changes in pulmonary function in children with asthma.

## 1 Introduction

Asthma is a heterogeneous respiratory disease that is associated with chronic inflammation. It is characterized by reversible airway obstruction, airway hyperresponsiveness, and airway remodeling, with cough, chest tightness, wheezing and shortness of breath as the main symptoms (1). Asthma seriously affects children's learning and their quality of life and is the second leading cause of death among chronic respiratory diseases (2). There is a diverse range of treatment options available to help patients improve their quality of life by reducing symptoms, managing asthma, and improving pulmonary functiony (3, 4). However, the current rate of asthma control is still unsatisfactory. Tosca et al. found that only 55% of patients had good control of asthma after treatment, 32.4% had partial control and 12.6% had no control (5). However, since asthma is a chronic airway inflammatory disease, it is particularly important to dynamically evaluate the therapeutic effect and level of control of chronic inflammation in the long term.

Currently, there are many clinical methods of evaluating the level of airway inflammation, including peripheral blood eosinophilic count, blood eosinophilic cationic protein level, induced sputum eosinophilic count, bronchial mucosal tissue biopsy, pulmonary function and fractional exhaled nitric oxide (FeNO) test (6–8). FeNO testing technology is widely used in the clinic because of its noninvasiveness, low requirement for patient cooperation and high repeatability. Combined determination of multiple exhaled nitric oxide (eNO) tests are crucial for comprehensive evaluation and more accurate individualized treatment of chronic airway inflammation. Recently, some studies have shown that FeNO in patients with asthma is significantly higher (9, 10), and FnNO in patients with allergic rhinitis (AR) is significantly higher than that in healthy children (11, 12). The coexistence of AR is one of the factors contributing to poor asthma control. Studies have shown that children with asthma and concomitant AR have higher levels of both FeNO and FnNO compared to those without AR. Moreover, asthma control status is negatively correlated with both FeNO and FnNO levels, with elevated FnNO indicating suboptimal asthma control (13). It is recommended to evaluate both upper and lower airway inflammation in combination with fractional nasally exhaled nitric oxide (FnNO). Combined measurement of FeNO and FnNO may help identify the presence of allergic rhinitis in patients with asthma, thereby predicting the risk of poor asthma control and guiding comprehensive management. In this study, we aimed to explore the clinical value of FeNO and FnNO and their relationship with asthma control level, as well as their correlation with pulmonary function, to provide a basis for a more comprehensive assessment of asthma control levels.

## 2 Material and methods

### 2.1 Study subjects

This study retrospectively analyzed the clinical data of 88 children with asthma who received treatment and follow-up at The First Hospital of Jilin University from 2019 to 2022. Participants were divided into two groups: a simple asthma group and an asthma combined with allergic rhinitis group. The children were divided into a rhinitis group and a non-rhinitis group based on whether they had comorbid allergic rhinitis. The non-rhinitis group consisted of 38 cases, and the rhinitis group consisted of 50 cases. All participating children underwent detailed medical history collection, physical examination, and relevant laboratory tests to confirm their diagnoses. This study was approved by the ethics committees of our research institutions (No. 2017-470). All participating patients and their parents or legal guardians agreed to be included in the study.

### 2.2 Inclusion and exclusion criteria

The inclusion criteria were as follows: (a) all patients met the criteria for diagnosis of bronchial asthma (14); (b) chronic persistent asthma was treated with standard protocols for more than 3 months; and (c) the patient was able to cooperate with lung ventilation function tests and FeNO and FnNO examination.

The exclusion criteria were as follows: (a) patients with congenital heart and lung diseases or cardiac insufficiency; (b) patients with chronic respiratory diseases; (c) patients with recent exacerbations of asthma; and (d) patients with incomplete clinical data or the inability to complete lung ventilation function tests or FeNO and FnNO examinations.

### 2.3 Data source

The clinical data of patients with bronchial asthma were retrospectively collected, including sex, age, height, weight, body mass index (BMI), FeNO level, FnNO level, Childhood Asthma Control Test (C-ACT) score and pulmonary function parameters. Patients were divided into the control group, partial control group and poor control group according to C-ACT scores, and FeNO and FnNO levels and pulmonary function parameters were compared among the three groups. We also analyzed whether FeNO and FnNO levels were correlated with various parameters of pulmonary function in patients with asthma.

### 2.4 Feno, FnNO and pulmonary function test

The FeNO and FnNO measurements were performed using the *Sunvou Medical Electronics NIOX VERO nitric oxide analyzer.* Prior to the measurements, the participants had followed the required preparations, *including* fasting from food and water, maintaining calm breathing, and avoiding strenuous exercise or intense crying. FeNO and FnNO determinations were conducted before pulmonary function tests, in accordance with the standards of t*he American Thoracic Society/European Respiratory Society* (15). FnNO testing employs the standardized nasal aspiration technique. The instrument performs constant-flow aspiration at 10 ml/s for 10 s, during which the subject activates a whistle to maintain oral expiratory pressure ≥10 cmH_2_O. Pulmonary function was measured by trained physicians using the J*aeger Master Screen Paed (Jaeger Company, Würzburg, Germany)*, in accordance with the standards of *the American Thoracic Society/European Respiratory Society* (16). The percentage predicted values (% pred) were calculated based on reference values for healthy Chinese children.

### 2.5 Asthma control level

The C-ACT is a Chinese version questionnaire (17, 18). The test consisted of seven questions—four questions for child response (parent's help in case of child needs help to read or understand) and three questions for parents. Result interpretation: After completing the test, the total score was calculated and analyzed by a doctor. The total score of the poor control group was ≤19 points, the partial control group was 20–23 points, and the control group was >23 points. The content is presented in Table 1.

**Table 1:** Control test for children (parent-reported).

Question		Score options	Points
1.	How is your child’s asthma today?	(0) Very bad; (1) Bad; (2) Good; (3) Very good	
2	Is asthma a problem when your child is playing, exercising, or running?	(0) Big problem—can't do what I want; (1) Problem—don't like it; (2) Small problem—can cope; (3) No problem	
3	Does your child cough because of asthma?	(0) All the time; (1) Most of the time; (2) Some of the time; (3) Never	
4	Does your child often wake up at night because of asthma?	(0) All the time; (1) Most of the time; (2) Some of the time; (3) Never	
5	During the past 4 weeks, how many days did your child have daytime asthma symptoms?	(0) Every day; (1) 19–24 days; (2) 11–18 days; (3) 4–10 days; (4) 1–3 days; (5) None	
6	During the past 4 weeks, how many days did your child wheeze during the day because of asthma?	(0) Every day; (1) 19–24 days; (2) 11–18 days; (3) 4–10 days; (4) 1–3 days; (5) None	
7	During the past 4 weeks, how many nights did your child wake up because of asthma?	(0) Every night; (1) 19–24 nights; (2) 11–18 nights; (3) 4–10 nights; (4) 1–3 nights; (5) None	
Total			

### 2.6 Statistical analysis

SPSS 23.0 was used for statistical analysis. Measurement data are expressed as the mean ± standard deviation, and analysis of variance was used for comparisons among multiple groups. Nonnormally distributed data are expressed as medians and interquartile ranges, and comparisons among groups were performed by the *Kruskal‒Wallis* test. The least significant difference (LSD) method was used to test the pairwise comparisons between groups. Statistical data are expressed as numbers (%), and the *χ*2 test was used for comparisons among groups. Spearman analysis was used for correlation analysis. *P* < 0.05 was considered statistically significant.

## 3 Results

### 3.1 Comparison of clinical data and pulmonary function of patients with asthma in rhinitis group and non-rhinitis group

A total of 88 patients with asthma were enrolled, including 50 (56.82%) in the rhinitis group, 38 (43.18%) in the non-rhinitis group. There were no significant differences in age, sex, height, weight, BMI and C-ACT between the two groups (*P* = 0.48, *P* = 0.42, *P* = 0.83, *P* = 0.22, *P* = 0.14, respectively, Table 2). The comparative analysis of FeNO and FnNO between the two groups showed that there was no significant difference (*P* = 0.80, *P* = 0.25, respectively, Table 2). And there was no significant difference in pulmonary ventilation function (FVC%, FEV1%, FEV1/FVC%, PEF%, MEF75%, MEF50%, MEF25%, MMEF%) between the two groups (*P* = 0.99, *P* = 0.58, *P* = 0.22, *P* = 0.65, *P* = 0.45, *P* = 0.45, *P* = *0.6*, respectively, Table 2).

**Table 2:** Comparison of clinical characteristics and pulmonary function of patients in rhinitis group and non-rhinitis group.

Parameters	Rhinitis group	Non-rhinitis group	*P* value
Height (m)	1.40 ± 0.17	1.38 ± 0.17	0.48
Age (years)	8.00 (6.75,10.00)	8.00 (6.00,10.00)	0.42
Weight (kg)	31.85 (23.82,50.08)	36.10 (24.20,47.60)	0.83
BMI (kg/m^2^)	16.57 (15.28,21.88)	18.55 (16.10,21.83)	0.22
Male, *n* (%)	37	24	0.275
C-ACT	18.50 (16.00,20.00)	20.00 (15.75,22.00)	0.14
FeNO	23.50 (16.75,36.00)	22.00 (18.00,32.25)	0.80
FnNO	375.50 (218.75,514.75)	312.50 (187.00,463.00)	0.25
FVC (%)	79.43 ± 20.68	79.38 ± 20.13	0.99
FEV_1_ (%)	75.63 ± 21.15	78.15 ± 21.50	0.58
FEV_1_/FVC (%)	95.11 ± 11.59	98.14 ± 11.30	0.22
PEF (%)	68.05 ± 21.38	70.13 ± 20.26	0.65
MEF_75%_ (%)	63.78 ± 23.78	67.63 ± 23.78	0.45
MEF_50%_ (%)	58.43 ± 26.74	62.96 ± 28.93	0.45
MEF_25%_ (%)	55.00 ± 32.81	58.68 ± 32.17	0.6

### 3.2 Comparison of clinical characteristics of patients in the poor control, partial control and control groups

A total of 88 patients with asthma were enrolled, including 43 (48.86%) in the poor control group, 34 (38.64%) in the partial control group and 11 (12.5%) in the control group. There were no significant differences in height, age, weight, BMI or sex among the three groups (*P* = 0.40, *P* = 0.26, *P* = 0.22, *P* = 0.31, *P* = 0.25, respectively, Table 3).

**Table 3:** Comparison of clinical characteristics of patients.

Clinical characteristics	Poor control group	Partial control group	Control group	*P* value
Number of patients (n)	43	34	11	
Male, *n* (%)	28 (65.12)	23 (67.65)	10 (90.91)	0.25
Age (years)	8.12 ± 1.72	7.94 ± 2.02	8.05 ± 1.98	0.26
Weight (kg)	31.00 (22.80, 47.70)	36.50 (24.38, 47.15)	42.60 (25.30, 65.30)	0.22
Height (m)	1.38 ± 0.18	1.39 ± 0.16	1.46 ± 0.18	0.40
BMI (kg/m^2^)	16.78 (15.39, 20.57)	17.19 (15.44, 22.96)	20.54 (15.39, 25.94)	0.31

### 3.3 Comparison of FeNO and FnNO levels and pulmonary function parameters in the poor control, partial control and control groups

Comparison of the FeNO and FnNO levels of the three groups revealed that the FeNO and FnNO levels in the poor control group were significantly increased (*P* < 0.05, Table 4). FeNO and FnNO levels decreased gradually with the improvement of asthma control, but the improvement between the partial control group and the control group was not significant.

**Table 4:** Comparison of FeNO and FnNO levels and pulmonary function parameters.

Parameters	Poor control group	Partial control group	Control group	*P* value
FeNO	26.0 (20.00, 38.00)	20.00 (16.75, 26.00)^a^	19.00 (16.00, 23.00)^a^	<0.05
FnNO	440.00 (221.00, 581.00)	272.50 (190.50, 385.00)^a^	232.00 (164.00, 394.00)^a^	<0.05
FEV_1_/FVC (%)	94.56 ± 10.66	98.88 ± 12.42	96.07 ± 11.34	0.26
PEF (%)	63.74 ± 20.79^c^	71.74 ± 19.91	80.69 ± 18.71^a^	<0.05
MEF_75%_ (%)	59.89 ± 24.25	69.52 ± 21.40	74.55 ± 25.15	0.08
MEF_50%_ (%)	52.20 ± 24.90^b^^,^^c^	65.51 ± 27.56^a^	76.55 ± 29.52^a^	<0.05
MEF_25%_ (%)	46.57 ± 30.98	62.08 ± 31.22	73.64 ± 36.81	0.07
MMEF (%)	49.81 ± 27.11^b^^,^^c^	65.04 ± 27.80^a^	70.54 ± 24.59^a^	<0.05

^a^ *p* < 0.05 compared with the poor control group.

^b^ *p* < 0.05 compared with the partial control group.

^c^ *p* < 0.05 compared with the control group.

Comparative analysis of lung ventilation function indicators among the three groups showed that the level of forced vital capacity (FVC) increased as asthma control improved, with a significant difference among the groups (Table 4, *P* < 0.01). Forced expiratory volume in 1 s (FEV_1_), maximal expiratory flow at 50% of the FVC (MEF_50%_) and maximal midexpiratory flow (MMEF) in the poor control group were significantly lower than those in the partial control group and the control group (Table 4, *P* < 0.01). The peak expiratory flow (PEF) of the poor control group was significantly lower than that of the control group (Table 4, *P* < 0.05). There were no significant differences in FEV_1_/FVC, MEF_75%_ and MEF_25%_ among the three groups (Table 4, *P* = 0.26, *P* = 0.08, *P* = 0.07, respectively).

### 3.4 Correlation of FeNO, FnNO and pulmonary function

The correlation analysis of FeNO, FnNO and various parameters of pulmonary function in patients with asthma showed that FeNO and FnNO had no significant correlation with pulmonary function parameters (Table 5, *P* = 0.27, *P* = 0.58, *P* = 0.48, *P* = 0.14, *P* = 0.11, *P* = 0.55, *P* = 0.93, *P* = 0.47).

**Table 5:** Correlation of FeNO, FnNO and pulmonary function parameters.

Parameters	FeNO		FnNO	
	*r*	*P*	*r*	*P*
FVC %	−0.104	0.34	0.118	0.27
FEV1 %	−0.16	0.14	0.06	0.58
FEV1/FVC %	−0.03	0.76	−0.077	0.48
PEF %	−0.029	0.79	0.159	0.14
MEF75 %	−0.005	0.97	0.174	0.11
MEF50 %	−0.112	0.30	0.07	0.55
MEF25 %	−0.177	0.10	−0.01	0.93
MMEF %	−0.126	0.24	0.078	0.47

## 4 Discussion

By analyzing FeNO and FnNO in patients with asthma, we found that FeNO and FnNO levels in patients with poorly controlled asthma were significantly higher than those in patients with partially controlled and well-controlled asthma. FEV_1_, MEF_50_ and MMEF decreased significantly in patients with poorly controlled asthma. We also analyzed the correlation among FeNO, FnNO and various parameters of pulmonary function in patients with asthma and found that there was no significant correlation among FeNO, FnNO and various pulmonary function parameters.

Research has shown that eNO, due to its high reactivity, contributes significantly to the pathophysiology of various lung conditions, particularly asthma (19). Inflammatory factors such as eosinophils, IgE and interleukin act on respiratory epithelial cells, resulting in elevated FeNO levels in asthmatic patients. The current assessment of asthma control is based on the Global Initiative for Asthma (GINA) guidelines for symptoms, reliever use, and activity limitations. Soto-Ramos et al. found that patients with well-controlled asthma had lower FeNO levels (20). However, Michils et al. found that changes in asthma symptoms did not coincide with changes in pulmonary function and FeNO (21). Our study found that FeNO levels differed according to the level of asthma control, with the highest level in the poor control group and a gradual decrease with improvement in asthma control. Therefore, our study suggested that the level of asthma control can be assessed by FeNO levels, and high FeNO levels may indicate poor control of asthma. Therefore, FeNO values can be used to assess asthma control more comprehensively.

Studies have shown that upper and lower respiratory tract diseases share common pathophysiological mechanisms, often coexist, and influence each other (22). The nasal passages and sinuses are also thought to be important sources of NO, and allergic rhinitis patients have elevated levels of NO in the nasal passages. Togias et al. found that allergic rhinitis symptoms are more common in patients with poorly controlled asthma (23). In this study, there were significant differences in FnNO in patients with different levels of asthma control, and FnNO in the poor control group was significantly higher than that in the partial control group and the control group, indicating that upper airway inflammation affects the level of asthma control. Therefore, attention should be given to evaluating the influence of upper airway inflammation on asthma control in children with unsatisfactory treatment results. The technical guidelines for exhaled biomarkers in lung disease advocate for the concurrent assessment of FeNO and FnNO to aid in the diagnosis and treatment of asthma and rhinitis (24). Both FeNO and FnNO levels were increased in patients with poorly controlled asthma, suggesting a persistent inflammatory state. Most cases of rhinitis in children are related to allergic factors, and a considerable proportion of children with AR will develop asthma. For children with asthma with high levels of FnNO, upper airway inflammation should be managed in conjunction with asthma treatment.

Pulmonary function plays an important role in the diagnosis of asthma, the severity of airway obstruction in asthma patients, and the evaluation of asthma control, which is an important auxiliary test in the diagnosis and treatment of asthma. Pulmonary function includes many parameters, the most widely used of which are FEV_1_, FEV_1_/FVC, PEF, MEF_75%_, MEF_50%_, MEF_25%_, and MMEF, which are used to evaluate the obstruction of small and large airways. The essence of asthma is chronic inflammation of the airways, and both large and small airways can be involved. A decrease in FEV_1_ and FEV_1_/FVC is the most common change in pulmonary function in patients with typical asthma, and most of these changes are combined with a decrease in small airway parameters. Our study grouped asthma patients based on C-ACT scores to further compare differences in pulmonary function between groups and found that the FVC%, FEV1%, MEF50% and MMEF% in the poor control group were significantly lower than those in the partial control group and the control group. Our study suggests that the symptoms and feelings of asthmatic patients are related to changes in pulmonary function, and attention should be given to the patients' own assessment of the disease in the process of diagnosis and treatment.

Chronic airway inflammation plays a key role in the pathogenesis of asthma, but whether poor pulmonary function is associated with severe inflammation is unclear. Although some studies have found an association between higher levels of inflammatory markers and more severe airflow restriction, a consensus has not been reached. Currently, the correlation between eNO and various parameters of pulmonary function is controversial. Habib et al. conducted a 1-year follow-up of 59 patients with asthma, and the results showed no correlation between FeNO and pulmonary function (25). Covar et al. found no correlation between FeNO and FEV_1_ and FEV_1_/FVC in children with asthma aged 6–17 years (26). However, one study reached a different conclusion. Jang et al. found that FeNO levels were negatively correlated with pulmonary function parameters to varying degrees (27). Zheng et al. conducted a study on the correlation among FeNO, FnNO and pulmonary function in patients with allergic asthma and nonallergic asthma and found that FnNO in the two groups was not correlated with any parameters of pulmonary function, while FeNO was negatively correlated with FVC, FEV_1_, FEV_1_/FVC, PEF, forced expiratory flow (FEF_25_), FEF_50_, FEF_75_ and MMEF (28). Our study found no correlation among FeNO, FnNO and various indicators of pulmonary function. Given the differences between the results of this study and those of previous studies, further research with large samples is needed.

There were several limitations in this study. First, the sample size in this study was small, and heterogeneity may exist. Further studies will be carried out in the future. Second, this was a single-center study. Large-scale multicenter studies with large samples are needed for further exploration. Third, the parents of patients with asthma had insufficient understanding and provided incomplete descriptions of patients' symptoms, which may have caused partial deviation in the C-ACT assessment. In the future, universal education of parents of asthma patients should be strengthened to conduct a more comprehensive and objective assessment of asthma.

## 5 Conclusions

In this study, FeNO and FnNO were examined in patients with asthma and found to be significantly increased in the poorly controlled asthma group, suggesting that for patients with asthma, simultaneous examination of FeNO and FnNO to assess the level of airway inflammation is beneficial to evaluate the level of asthma control. However, our study also found no significant correlation among FeNO, FnNO and various parameters of pulmonary function.

## Data Availability

The original contributions presented in the study are included in the article/Supplementary Material, further inquiries can be directed to the corresponding author.
